# Trop2 enhances invasion of thyroid cancer by inducing MMP2 through ERK and JNK pathways

**DOI:** 10.1186/s12885-017-3475-2

**Published:** 2017-07-14

**Authors:** Hongyu Guan, Zejun Guo, Weiwei Liang, Hai Li, Guohong Wei, Lijuan Xu, Haipeng Xiao, Yanbing Li

**Affiliations:** Department of Endocrinology and Diabetes Center, The First Affiliated Hospital of Sun Yat-sen University, 58 Zhongshan Road II, Guangzhou, Guangdong 510080 China

**Keywords:** Trop2, Thyroid cancer, Invasion, MMP2, MAPK

## Abstract

**Background:**

Mounting evidence has showed that Tumor-associated calcium signal transducer 2 (Trop2) is upregulated in various kinds of human cancers and plays important roles in tumorigenesis. However, the expression status and functional significance of Trop2 in thyroid cancer are largely unknown.

**Methods:**

We first determined the expression of Trop2 by using RNAseqV2 data sets for thyroid cancer deposited on The Cancer Genome Atlas (TCGA) website. The expression of Trop2 was then confirmed by real-time reverse transcription-polymerase chain reaction (RT-PCR) and immunohistochemistry assays. Cell invasion and migration were assessed by conducting Transwell and wound healing assays. Furthermore, we explored the underlying mechanisms by using real-time RT-PCR, Western blot, zymography, and luciferase reporter assays.

**Results:**

In this study, we demonstrated that the expression of Trop2 was significantly elevated in thyroid cancer and that its expression level was correlated with the tumor-node-metastasis (TNM) staging and N classification. Dysregulation of Trop2 altered the invasive capability of thyroid cancer cells. Further mechanistic study revealed that MMP2 expression was upregulated by Trop2. Moreover, we found that the effects of Trop2 were dependent on ERK and JNK pathways. The results from clinical specimens showed that Trop2 expression correlated with MMP2 expression in primary thyroid cancer.

**Conclusion:**

The current study suggests that elevated expression of Trop2 may represent an important molecular hallmark that is biologically and clinically relevant to the progression of thyroid cancer.

## Background

The incidence of thyroid cancer, the most common endocrine malignancy, continues to increase steadily during the past few decades worldwide [1, 2]. The majority of thyroid cancer types are classified as: follicular epithelial cell-derived papillary thyroid cancer (PTC), follicular thyroid cancer (FTC), anaplastic thyroid cancer (ATC), and para-follicular C-cell derived medullary thyroid cancer (MTC) [3]. The prognosis of patients with thyroid cancer is closely correlated with local invasion outside the thyroid capsule and the development of distant metastases [4]. Therefore, dissecting the molecular mechanisms underlying thyroid cancer invasion and metastasis is still imperative and may put new insight into the clinical treatment of thyroid cancer.

Human tumor-associated calcium signal transducer 2 (TACSTD2), also known as trophoblast cell-surface antigen 2 (Trop2), is a type I transmembrane glycoprotein originally identified in human placental trophoblastic tissue [5]. As a cell surface receptor, it can recognize specific ligands [6] and increase the level of intracellular calcium [7]. Although the biological function of Trop2 is unclear, accumulating evidence has demonstrated that its expression is elevated in various malignant tissues, whereas in human normal tissues relatively low or no Trop2 expression is observed [8–11]. Over-expression of Trop2 has been implicated to correlate with poor prognosis of patients with several kinds of cancers [12, 13]. Immunohistochemistry (IHC) was performed in 94 PTC specimens and results showed that Trop2 was over-expressed in PTC [14]. However, the expression of Trop2 has not been characterized in FTC and ATC. Moreover, the clinical and functional significance of Trop2 in thyroid cancer remains unclear.

MAPK (mitogen-activated protein kinase) signaling pathway is a highly conserved intracellular pathway that plays vital roles in transmission of signals to cell nucleus, where they transcriptionally regulate genes that are involved in various cellular processes [15, 16]. The fundamental role of MAPK signaling pathway has been well demonstrated in human tumorigenesis, particularly for PTC [17]. Recent studies, which focused on investigating the molecular background of thyroid cancer, have shown that inappropriate activation of MAPK is a vital intracellular regulator for thyroid tumorigenesis [18]. The aberrant activation of MAPK pathway in thyroid cancer is driven by some genetic alterations, including BRAF, RAS, RET-PTC, and ALK mutations [19–22] . To fully understand the regulation of MAPK signaling in thyroid cancer is biologically as well as clinically important for future development of treatment strategies.

In the current study, we aimed at investigating the role of Trop2 in the development and progression of thyroid cancer. We evaluated the pro-invasive effect of Trop2 in thyroid cancer and explored the possible underlying mechanisms.

## Methods

### Cell lines and reagents

The K1 (papillary cancer), FTC-133 (follicular cancer), and 8505C (anaplastic cancer) thyroid cancer cell lines were from the European Collection of Cell Cultures (ECACC, Salisbury, United Kingdom). Cell lines were cultured in Dulbecco’s Modified Eagle Medium (DMEM, Life Technology, Grand Island, NY) supplemented with 10% fetal bovine serum (FBS, Gibco, Grand Island, NY) in a humidified cell culture incubator at 37 °C and 5% CO_2_ . PD98059 (ERK1/2 inhibitor), SP600125 (JNK inhibitor), and SB203580 (p38 inhibitor) were purchased from Cell Signaling Technology (Beverly, MA).

### Patients and tissue specimens

This study was conducted on a total of 96 cases of paraffin embedded thyroid carcinomas (67 cases of PTC, 20 cases of FTC, and 9 cases of ATC), 13 cases of goiters, and 15 cases of adenoma samples, which had been clinically and histologically diagnosed at the First Affiliated Hospital of Sun Yat-sen University between 2010 to 2015. Eighteen freshly collected thyroid cancer specimens and the matched adjacent non-cancerous thyroid tissues were collected, frozen, and stored in liquid nitrogen until assayed. Informed consent from patients and ethics approval from the Institutional Research Ethics Committee was obtained.

### Immunohistochemistry

Immunohistochemistry (IHC) analysis was performed to study altered protein expression in formalin-fixed and paraffin-embedded human thyroid lesions. The following primary antibodies were used: anti-Trop2 (R&D Systems, Minneapolis, MN), and anti-MMP2 (Lifespan Bioscience, Seattle). The degree of immunostaining was examined and scored independently by two observers by combining both the proportion of positively staining tumor cells and the staining intensity as previously described [23].

### Vectors and retroviral infection

Trop2 construct was generated by sub-cloning PCR-amplified full-length human Trop2 cDNA into pQCXIP (Clontech, Mountain View, CA). The Trop2-shRNA clones (TR308966) were purchased from Origene (Rockville, MD, USA). PT67 (Clontech, Mountain View, CA), an NIH 3 T3-derived packaging cell line, was purchased for retroviral transduction [24]. The indicated plasmids were transfected into the PT67 cell respectively using Lipofectamine 3000 (Invitrogen, San Diego, CA). The supernatant was harvested, passed through a 0.45 μm filter, and incubated with indicated cells together with 8 μg/mL polybrene. Stable cell lines were selected by treatment with 0.5 μg/mL puromycin for 10 days, beginning 48 h after infection.

### Western blotting

Western blotting (WB) was performed according to a standard method as described previously [25]. The following primary antibodies were used: anti-Trop2 (R&D Systems, Minneapolis, MN), anti-ERK1/2 and anti-phospho-ERK1/2 (Cell Signaling Technology, Beverly, MA), anti-p38, anti-phospho-p38, anti-JNK, and anti-phospho-JNK (Abcam, Cambridge, MA), and anti-α-tubulin (Sigma-Aldrich, St. Louis, MO).

### RNA extraction and real-time polymerase chain reaction (PCR)

RNA extraction, RT, and real-time PCR were performed as described previously [25]. The primers selected are as follows: MMP2 forward, 5′-CCAGCAAGTAGATGCTGCCT-3′ and reverse, 5′-GGGGTCCATTTTCTTCTTCA-3′; MMP7 forward, 5′-CACATCAGTGGGAACAGGC-3′ and reverse, 5′-GCATTTCCTTGAGGTTGTCC-3′; MMP9 forward, 5′-AGACGACATAGACGGCATCC-3′ and reverse, 5′-CTGTCGGCTGTGGTTCAGT-3′; MMP11 forward, 5′- CCACTGACTGGAGAGGGGT-3′ and reverse, 5′- TTCACAGGGTCAAACTTCCA-3′; MMP13 forward, 5′- TGATGAAACCTGGACAAGCA-3′ and reverse, 5′- GGTCCTTGGAGTGATCCAGA-3′; and glyceraldehyde-3-phosphate dehydrogenase (GAPDH) forward, 5′-GACTCATGACCACAGTCCATGC-3′ and reverse, 5′-AGAGGCAGGGATGATGTTCTG-3′. Data analysis was performed using the comparative Ct method and results were normalized to GAPDH. Each sample was analyzed three times in triplicate.

### Transwell assay

Cells (2 × 10^4^) in serum-free medium were plated into the upper chamber of 8 μm pore boyden chambers coated with or without Matrigel (BD Biosystems, San Jose, CA). The lower chamber was filled with medium containing 10% FBS. After 24 h incubation, cells on the surface of upper chamber were removed by scraping with a cotton swab. Migrating and invading cells on the lower membrane surface were fixed in 4% paraformaldehyde, stained with 0.1% crystal violet and counted in 5 random fields. Each assay was replicated three times.

### Wound healing assay

When indicated cells reached 90-95% confluence, they were scratched with a micropipette tip in the cell monolayer. After 24 h incubation, recovery of the wound was observed and images were captured by a phase-contrast microscope.

### Gelatin zymography analysis

Enzymatic activity of MMP2 was detected using MMP Zymography Assay Kit (P1700, Applygen Technologies, Beijing, China). Briefly, supernatants of indicated cells were collected and BCA assay measure was performed to determine the protein content. Equal amount of supernatants were mixed with an equal volume of 2 × SDS-PAGE non-reducing buffer, and electrophoresed on 8% polyacrylamide gels containing 1 × substrate G. The gels were then rinsed by 1 × Buffer A twice at room temperature and then incubated in 1 × Buffer B at room temperature for 4 h. The gels were then stained with Coomassie blue R250 and then destained in destaining buffer (10% acetic acid and 20% methanol). Each assay was replicated three times.

### Luciferase reporter assay

The reporter plasmid pGL2-MMP2 was purchased from Addgene (Cambridge, MA). The AP1 reporter assay kit was purchased from SABiosciences (Frederick, MD). Dual-Luciferase reporter assays were performed according to the manufacturer’s instructions (Promega, Madison, WI) and as described previously [25].

### AP1 decoy oligodeoxynucleotide (ODN)

AP1 decoy and mutated control used were double-stranded phosphorothioate-oligonucleotides. The AP-1 decoy OND sequence was 5′-CGCTTGATGACTCAGCCGGAA-3′; the mutated control was 5′- CGCTTGATGACTTGGCCGGAA-3′. Double strands (ds) ODN were prepared by melting complementary OND at 95 °C for 5 min and subsequently at RT for 4 h [26].

### Statistical analysis

All statistical analyses were carried out using the SPSS 13.0 statistical software package. All values represent at least three independent experiments and are expressed as the means ± SD. The differences between two experimental conditions were compared on a one-to-one basis using the Student’s t tests. Spearman rho correlation method was used to describe correlations between expression data. *P* < 0.05 was considered statistically significant.

## Results

### Trop2 is overexpressed in thyroid cancer

**Fig. 1:**
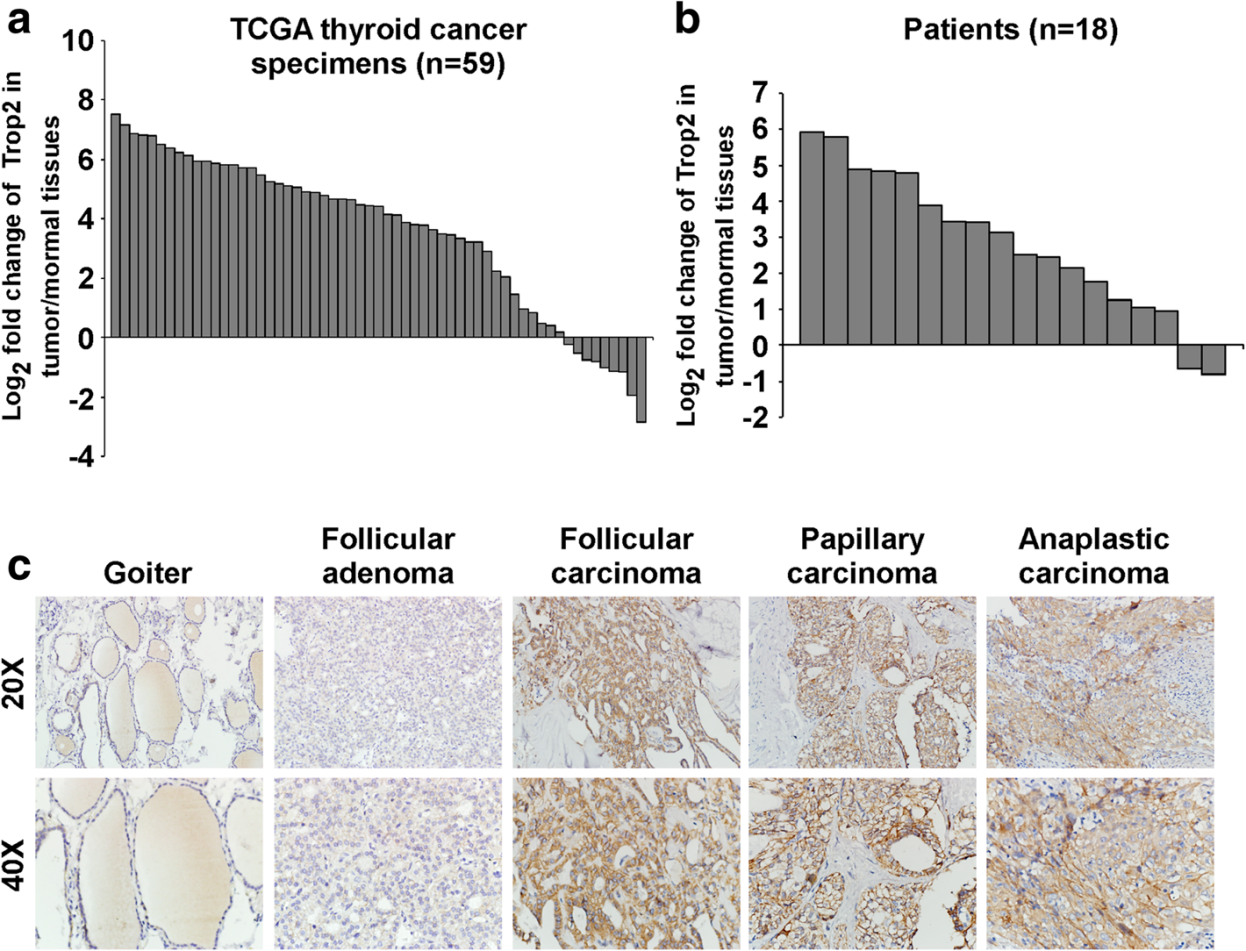
Trop2 is overexpressed in thyroid cancer. **a** The expression of Trop2 in 59 pairs of primary tumors versus paired non-tumorous thyroid tissues using RNAseqV2 data sets deposited on TCGA website. **b** Expression of Trop2 in 18 paired tumors and adjacent non-tumorous thyroid tissues assessed by qRT-PCR. Experiments were repeated for three times in triplicate. **c** Representative images of IHC assays on Trop2 expression in thyroid lesions

To explore the expression of Trop2 in thyroid cancer, we initially investigated the expression of Trop2 in 59 pairs of thyroid tumors and their adjacent non-tumorous thyroid tissues using RNAseqV2 data sets for thyroid cancer deposited on The Cancer Genome Atlas (TCGA, https://cancergenome.nih.gov/) website. The results showed that Trop2 expression level was significantly elevated in most thyroid cancer tissues as compared with their respectively adjacent non-tumorous thyroid tissues (Fig. 1a). Next, we validated the expression of Trop2 in 18 pairs of tumors and adjacent non-tumorous tissues using qRT-PCR. As shown in Fig. 1b, in agreement with the TCGA data, the expression levels of Trop2 were significantly increased in most thyroid tumor tissues in comparison with those in adjacent non-tumorous thyroid tissues. We next investigated the clinical relevance of Trop2 in thyroid cancer, we performed IHC to analyze the expression levels of Trop2 in goiters (13 cases), adenomas (15 cases), and thyroid cancers (96 cases). As compared with benign thyroid lesions, namely, goiters and adenomas, the expression level of Trop2 was significantly upregulated in thyroid cancer tissues (Fig. 1c). Intriguingly, in most goiters (10/13) and adenoma tissues (12/15), the expression of Trop2 was low, on contrary, Trop2 is highly expressed in 53.1% of thyroid malignant lesions (51/96). Importantly, significant correlations between Trop2 expression and tumor-node-metastasis (TNM) staging (*p* = 0.041) or N classification (*p* = 0.013) were observed (Table 1). Taken together, in accordance with previous studies, these data suggest that Trop2 is upregulated and may play functional roles in thyroid cancer.

**Table 1:** Association between clinicopathologic parameters and level of Trop2 protein expression in thyroid cancer patients

Clinicopathologic variables	Trop2		*P* value
	Low	High	
Age			
< 45 yr.	24	25	0.673
≥ 45 yr.	21	26	
Gender			
Male	10	13	0.708
Female	35	38	
TNM stage			
I and II	36	31	0.041
III and IV	9	20	
T classification			
T1 and T2	38	40	0.451
T3 and T4	7	11	
N classification			
N0	30	21	0.013
N1	15	30	

### Depletion of endogenous Trop2 inhibits the invasion and migration of thyroid cancer cells

**Fig. 2:**
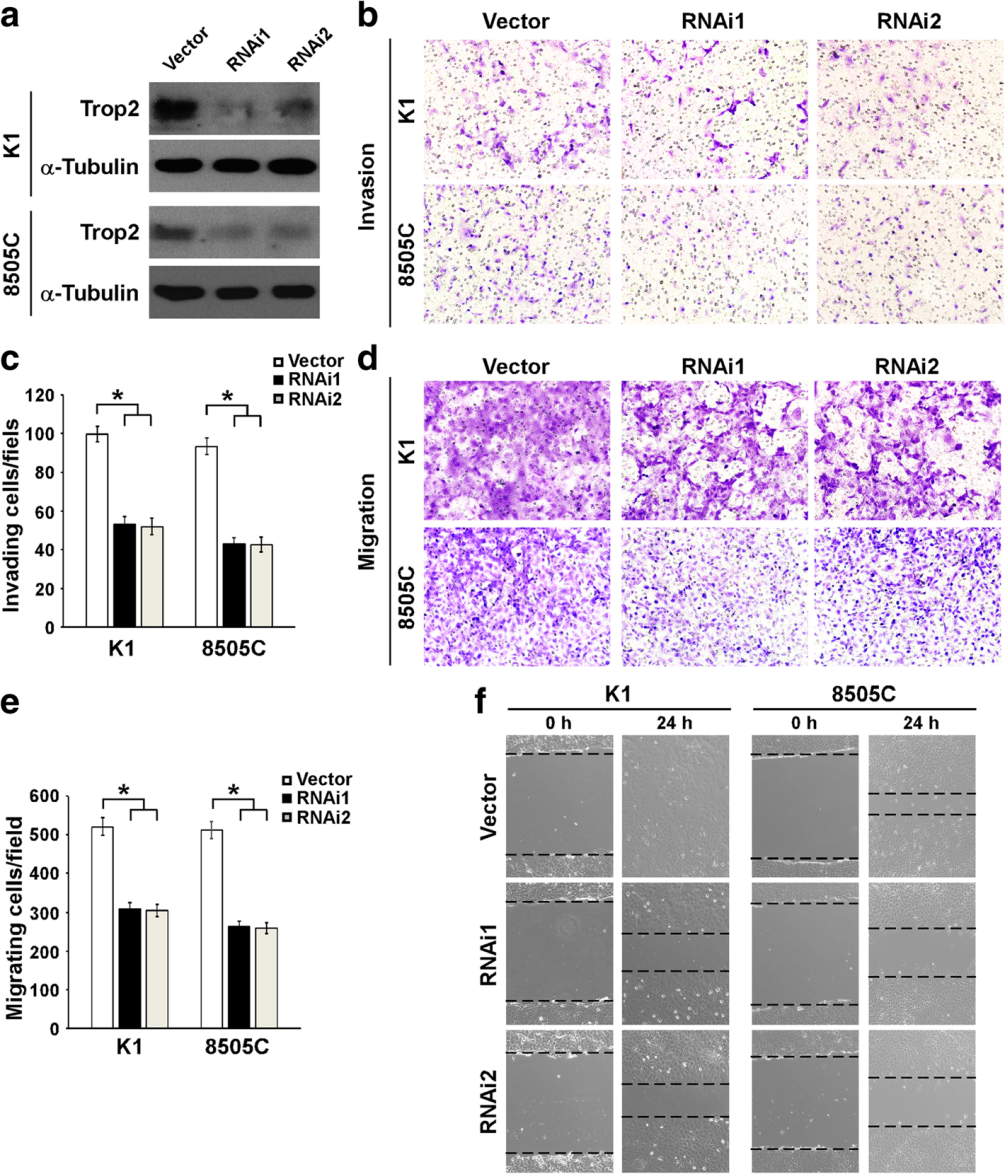
Knockdown of endogenous Trop2 suppresses the invasion and migration of thyroid cancer cells. **a** Trop2 knockdown was achieved by introducing specific shRNAs in thyroid cancer cells. The protein levels of Trop2 in indicated cells were assessed by WB. α-tubulin was used as a loading control. **b** Silencing of Trop2 led to significant decrease of invasive capability of thyroid cancer cells. The indicated cells traveled through the membrane by Transwell invasion assay (coated with Matrigel) were stained with crystal violet and imaged using a microscope. **c** The histogram showed the invading cells per field. The data are reported as mean ± SD of three independent experiments. *, *P* < 0.05. **d** Representative images of Transwell migration assay (coated without Matrigel) results of indicated cells. **e** Diagram of Transwell migration assay of indicated cells. The data are reported as mean ± SD of three independent experiments. *, *P* < 0.05. **f** Wound healing analyses of indicated cells. Streaks were created with a tip, and the representative phase-contrast images of the extent of cell migration into the wounded area at 24 h are shown. Experiments were repeated for three times

Given that it has been demonstrated that Trop2 is associated with the invasion and metastasis of several human cancers [27, 28], we next investigated the role of Trop2 in regulating thyroid cancer cell invasion and migration. To this end, we silenced endogenous Trop2 by specific short hairpin (shRNA) in K1 and 8505C cells. The effect of knockdown was confirmed by performing WB analyses (Fig. 2a). We next investigated whether knockdown of Trop2 plays roles in cell invasion and migration. Results of Transwell invasion (coated with Matrigel) and migration (without Matrigel) assays showed that knockdown of endogenous Trop2 suppressed the invasiveness and migration of K1 and 8505C cells as compared with control cells (Fig. 2b-e). Furthermore, migration capabilities of indicated cells were assessed by wound healing assay. As shown in Fig. 2f, depletion of Trop2 expression in thyroid cancer resulted in retarded wound closing when compared with the vector control cells. Taken together, these results suggest that silencing of the Trop2 inhibits the invasiveness and migration capability of thyroid cancer cells.

### Ectopic over-expression of Trop2 promotes the invasion and migration of thyroid cancer cells

**Fig. 3:**
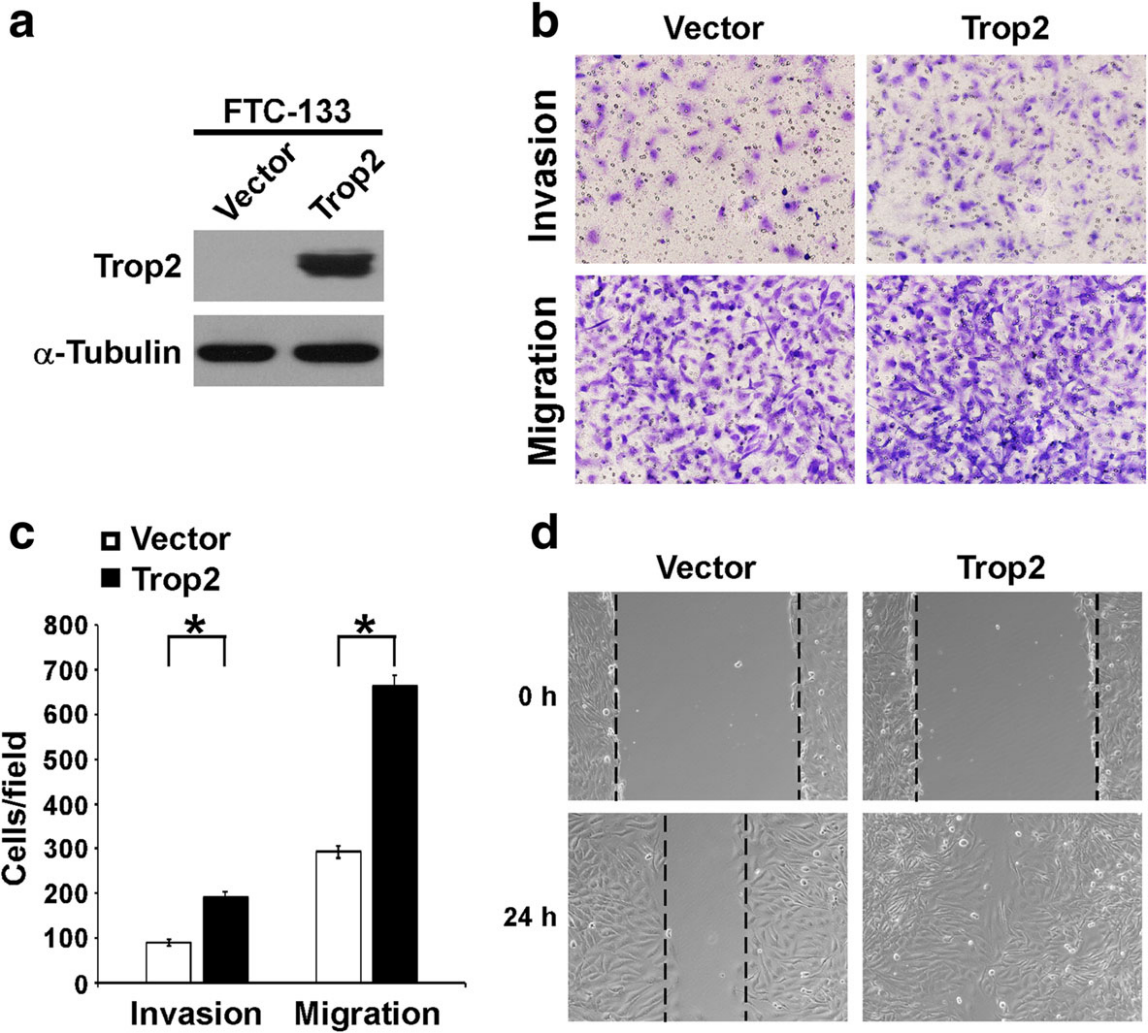
Over-expression of Trop2 promotes the invasion and migration of thyroid cancer cells. **a** Overexpression of Trop2 in FTC-133 thyroid cancer cells was analyzed. The protein levels of Trop2 in indicated cells were assessed by WB and α-tubulin was used as a loading control. **b** Overexpression of Trop2 increased the invasive (with Matrigel) and migratory (without Matrigel) capability of FTC-133 cells. **c** The histogram showed the invading and migrating cells per field. The data are reported as mean ± SD of three independent experiments. *, *P* < 0.05. **d** Wound healing analyses of indicated cells. Streaks were created with a tip, and the representative phase-contrast images of the extent of cell migration into the wounded area at 24 h are shown. Experiments were repeated for three times

In order to further investigate the role of Trop2 in thyroid cancer cell invasion and migration, Trop2 was stably transduced into FTC-133 cells, in which the expression of Trop2 cannot be detected (Fig. 3a). As shown in Fig. 3, b and c, Matrigel-coated or uncoated Transwell assays indicated that Trop2 over-expression significantly enhanced the ability of invasiveness and migration of thyroid cancer cells. In addition, wound healing assay showed that over-expression of Trop2 promoted the migratory speed of FTC-133 cells as compared with vector control cells (Fig. 3d). These data, together with the results from Fig. 2, indicate that Trop2 is involved in the invasion and migration of thyroid cancer cells.

### Trop2 regulates the expression of MMP2

**Fig. 4:**
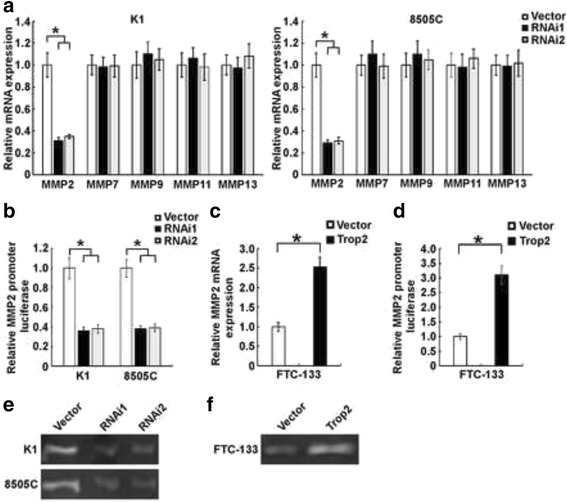
Trop2 regulates the expression of MMP2. **a** Silencing of Trop2 in K1 and 8505C thyroid cancer cells resulted in down-regulation of MMP2 expression, but not MMP7, MMP9, MMP11, and MMP13 expression. The data are reported as mean ± SD of three independent experiments. *, *P* < 0.05. **b** Depletion of Trop2 in K1 and 8505C thyroid cancer cells resulted in inhibition of the MMP2 promoter reporter activity. The data are reported as mean ± SD of three independent experiments. *, *P* < 0.05. **c** Ectopic overexpression of Trop2 promotes the expression of MMP2 in FTC-133 thyroid cancer cells. The data are reported as mean ± SD of three independent experiments. *, *P* < 0.05. **d** Trop2 overexpression enhanced the MMP2 promoter reporter activity. The data are reported as mean ± SD of three independent experiments. *, *P* < 0.05. **e** Conditioned media were prepared by incubating vector-control or Trop2-knockdown cells in serum-free media for 24 h. MMP2 activities are analyzed by gelatin zymography. **f** Gelaltin zymography analysis of serum-free conditioned medium from FTC-133-vector, FTC-133-Trop2 cells

It has been well demonstrated that matrix metalloproteinases (MMPs) play important role in tumor progression. Many MMP family members, such as MMP2, MMP7, MMP9, MMP11, and MMP13, have been implicated to be associated with the development and progression of thyroid cancer [29]. In light of these, we therefore investigated whether MMPs were involved in the effects of Trop2 on thyroid cancer cell invasion. As shown in Fig. 4a, only mRNA level of MMP2, not MMP7, MMP9, MMP11 or MMP13, was suppressed by Trop2 depletion. Promoter assay showed that knockdown of Trop2 decreased the luciferase activity of MMP2 promoter in K1 and 8505C cells (Fig. 4b). To further confirm the role of Trop2 in thyroid cancer, we evaluated the effects of over-expressing Trop2 on the MMP2 expression in FTC-133 cells. As expected, ectopic over-expression of Trop2 significantly promoted the expression and promoter activity of MMP2 in FTC-133 cells (Fig. 4c and d). Moreover, zymography were performed to test the MMP2 enzymatic activity in Trop2 dysregulated thyroid cancer cells. As shown in Fig. 4e and f, knockdown of Trop2 in K1 and 8505 cells reduced the MMP2 activity, whereas, overexpression of Trop2 in FTC-133 cells resulted in increased activity of MMP2. Collectively, our data suggest that the role of Trop2 on thyroid cancer cell invasion and migration was mediated by regulation of MMP2.

### AP1 mediates the regulatory role of Trop2 on MMP2 expression

**Fig. 5:**
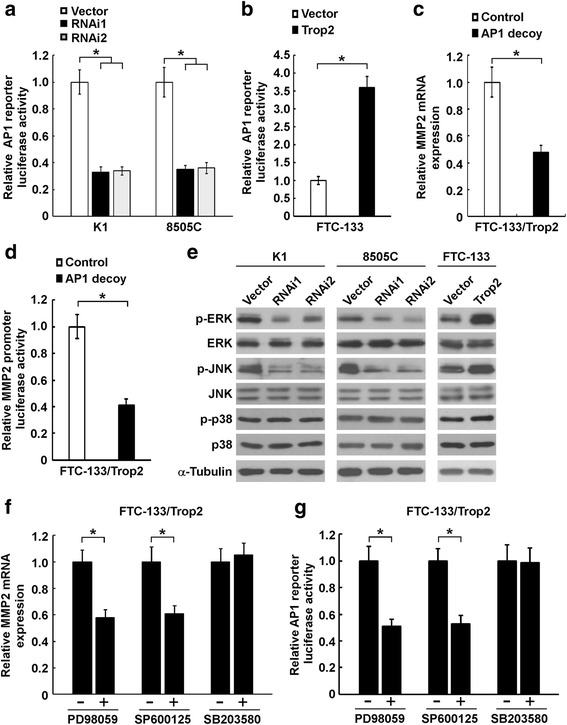
ERK/JNK/AP1 signaling mediates the effect of Trop2 on MMP2 expression. **a** Comparison of AP1 reporter activity in indicated cells. The luciferase reporter showed decreased activation of AP1 reporter in Trop2 knocked-down K1 and 8505C cells. The data are reported as mean ± SD of three independent experiments. *, *P* < 0.05. **b** Overexpression of Trop2 promoted the AP1 reporter activity. The data are reported as mean ± SD of three independent experiments. *, *P* < 0.05. **c** and **d** AP1 ODNs significantly inhibited the effects of Trop2 on MMP2 expression (**c**) and MMP2 promoter reporter activity (**d**). The data are reported as mean ± SD of three independent experiments. *, *P* < 0.05. **e** WB analysis showed that deregulated expression of Trop2 significantly altered the phosphorylation of ERK and JNK. **f** and **g** Cells were treated with a concentration of 10 μM PD98059, 10 μM SP600125, or 10 μM SB203580 for 24 h. After incubation, MMP2 mRNA level was determined by qRT-PCR (**f**) and AP1 reporter activity was assessed by Dual-Luciferase reporter assays (**g**). The data are reported as mean ± SD of three independent experiments. *, *P* < 0.05

To investigate the signaling pathway that controls the expression of MMP2 in response to alteration of Trop2 expression, the activation status of transcription factor AP1, a well-known regulator of MMP2, was evaluated in thyroid cancer cells with Trop2 overexpression or knockdown. To this end, we first examined whether dysregulated expression of Trop2 has an effect on AP1 transcriptional activity. As shown in Fig. 5a and b, the luciferase activity of AP1 reporter was decreased by depletion of Trop2 and increased when Trop2 was overexpressed, suggesting a potential role of AP1 in the signaling cascade that mediates the regulatory role of Trop2 on MMP2 expression. To further confirm the critical role of AP1 in this course, AP1 decoy phosphorothioated double-stranded ONDs were used. As indicated in Fig. 5c, the Trop2-stimulated MMP2 mRNA expression was suppressed by the decoy of AP1. Consistently, AP1 decoy also lead to inhibition of Trop2-induced MMP2 promoter reporter activity (Fig. 5d). Together, these data indicate that AP1 is critically required for Trop2-regulated MMP2 expression.

### Trop2 regulates MMP2 expression by modulating ERK and JNK signaling

Next, we delineated how Trop2 regulates the activity of AP1. As shown in Fig. 5e, the phosphorylation levels of ERK1/2 and JNK were significantly decreased in Trop2-knocked down thyroid cancer cells as compared with vector control cells, and increased phosphorylation of ERK1/2 and JNK was observed in Trop2-transduced cells. However, the phosphorylation of p38 did not change when the expression of Trop2 was deregulated (Fig. 5e). To further confirm the role of MAPK pathway in Trop2-induced MMP2 expression, the MAPKs inhibitors, namely PD98059 (ERK1/2 inhibitor), SP600125 (JNK inhibitor), and SB203580 (p38 inhibitor) were used. Among these inhibitors, only PD98059 and SP600125 could suppress Trop2-induced MMP2 expression, suggesting that ERK1/2 and JNK play a critical role in Trop2-regulated MMP2 expression (Fig. 5f). Moreover, Trop2-induced activation of AP1 was significantly inhibited by treatment with PD98059 or SP600125 (Fig. 5g). Taken together, these results suggest that MAPK ERK and JNK signaling are involved in the regulatory roles of Trop2 on AP1 activation and MMP2 expression.

### Trop2 expression correlates with MMP2 expression in primary thyroid cancer

**Fig. 6:**
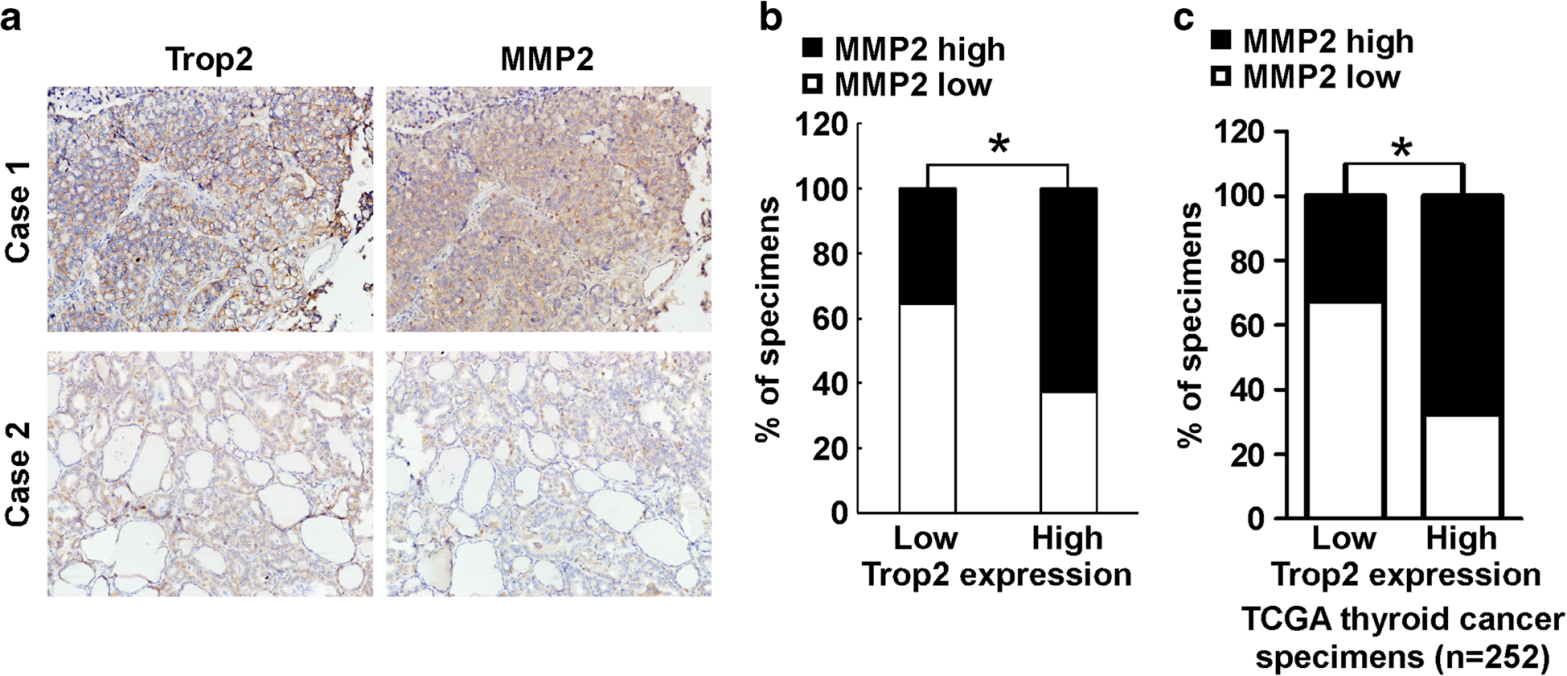
Trop2 expression correlates with MMP2 expression in primary thyroid cancer. **a** Expression of Trop2 is associated with MMP2 expression levels in clinical thyroid cancer specimens. Two representative cases are shown. **b** Percentage of specimens showing low or high Trop2 expression in relation to the expression levels of MMP2. *, *P* < 0.05. **c** The association of Trop2 and MMP2 in 252 cases of thyroid cancer specimens were analyzed using RNAseqV2 data sets deposited on TCGA website. *, *P* < 0.05

We next investigate whether there is an association between the expression levels of Trop2 and MMP2 in the clinical specimens. As shown in Fig. 6a and b, 62.7% (32 cases) of thyroid cancer samples with high Trop2 expression (51 cases) exhibited high levels of MMP2, whereas 64.4% (29 cases) of samples with low Trop2 expression (45 cases) showed low levels of MMP2 (*p* < 0.05) (Spearman rho = 0.276, *P* = 0.007). Furthermore, we used RNAseqV2 data mined from TCGA and sorted the patient cohort into the top and bottom quartiles of Trop2 expression (Trop2 high and low, *n* = 126 respectively) (Spearman rho = 0.300, *P* < 0.001). As shown in Fig. 6c, 67.5% (85 cases) of samples with low Trop2 expression exhibited low levels of MMP2 (less than the median), whereas 32.5% (41 cases) of samples with high Trop2 expression showed low levels of MMP2, suggesting that Trop2 expression significantly correlated (*P* < 0.05) the level of MMP2 in thyroid cancer. These findings are consistent with Trop2 functioning as a regulator of MMP2 expression.

## Discussion

As an oncogene, Trop2 has been found to be up-regulated in various kinds of human cancers, including glioma, breast cancer, cervical cancer, colorectal cancer, ovarian cancer, pancreatic cancer, and prostate cancer [9, 30–32]. In line with previous studies [14], the results of current study showed that the expression of Trop2 is overexpressed in clinical thyroid cancer specimens. Notably, the expression of Trop2 is correlated with TNM staging and N classification of thyroid cancer. These data put new insights into the molecular alterations that are involved in the thyroid tumorigenesis. Further studies are needed to clarify the mechanisms underlying the overexpression of Trop2 in thyroid cancer, which will enhance our understanding of the pathogenesis of thyroid malignancy.

Previous studies about the biological roles of Trop2 in cancers were mainly focused on the pro-proliferative effect of Trop2 in human cancers [31–33]. Nevertheless, a few studies have assessed the pro-invasive role of Trop2 in tumorigenesis [12, 34]. Consistent with these studies, our data showed that the invasion and migration ability of thyroid cancer was inhibited by depletion of Trop2 expression and promoted when Trop2 was over-expressed, suggesting a requirement of Trop2 in thyroid cancer invasion and migration.

Accumulating evidence indicate that MMPs play important roles in tumor development and progression [35]. Given that ECM is the first vital barrier in the tumor invasion and metastasis process and the main component of ECM is type IV collagen, together with important role of MMP2 in degradation of type IV collagen, MMP2 is regarded as a key enzyme involved in invasion and metastasis of cancer [36]. Trop2 seems to be critical in regulating MMP2 expression in our study because our data revealed upregulation of MMP2 mRNA level and activation of MMP2 promoter reporter in Trop2-overexpressing thyroid cancer cells, as well as down-regulation of MMP2 mRNA and inactivation of MMP2 promoter reporter in Trop2 knocked down thyroid cancer cells. The results of our current study using AP1 decoy ODNs confirmed that activation of AP1 was attributable to regulation of MMP2. Therefore, data obtained from the present study suggest that AP1 may play a role in mediating the regulation of MMP2 by Trop2.

In current study, we showed that expression of Trop2 in thyroid cancer has effect on MAPK activation. These results provide new insights into the complex regulatory mechanism for activation of MAPK in thyroid cancer. Nevertheless, mechanism by which Trop2 activates MAPK in thyroid cancer cells remains to be determined.

## Conclusions

In summary, we show that Trop2 is overexpressed in thyroid cancer and promotes the invasion and migration of thyroid cancer cells via MAPK ERK/JNK/AP1/MMP2 signaling, potentially offering new molecular targets for treatment of thyroid cancer.

## Abbreviations

ATC: Anaplastic thyroid cancer
FTC: Follicular thyroid cancer
IHC: Immunohistochemistry
MAPK: Mitogen-activated protein kinase
MMPs: Matrix metalloproteinases
ODN: Oligodeoxynucleotide
PTC: Papillary thyroid cancer
RT-PCR: Real-time reverse transcription-polymerase chain reaction.
TCGA: The Cancer Genome Atlas
TNM: Tumor-node-metastasis
Trop2: Tumor-associated calcium signal transducer 2
WB: Western blotting
